# Spectral dataset of natural objects’ reflectance from the Southern cone of South America

**DOI:** 10.1038/s41597-025-04675-9

**Published:** 2025-04-09

**Authors:** Agustín Gutiérrez, Bárbara Silva, José M. Fanchini, Takuma Morimoto, Pablo A. Barrionuevo, María L. Sandoval-Salinas

**Affiliations:** 1 Instituto de Investigación en Luz, Ambiente y Visión (ILAV), Universidad Nacional de Tucumán (UNT) - Consejo Nacional de Investigaciones Científicas y Técnicas (CONICET), Tucumán, Argentina; 2 Department of Experimental Psychology, University of Oxford, Oxford, UK; 3 Allgemeine Psychologie, Justus-Liebig-Universität Gießen, Gießen, Germany; 4 Allgemeine und Biologische Psychologie, Philipps-Universität Marburg, Marburg, Germany; 5 Instituto de Investigaciones de Biodiversidad Argentina (PIDBA), Facultad de Ciencias Naturales e Instituto Miguel Lillo, Universidad Nacional de Tucumán (UNT), Tucumán, Argentina

**Keywords:** Evolutionary ecology, Colour vision, Natural variation in plants

## Abstract

The reflection in natural objects mediates an important fraction of the light reaching animal photoreceptors. Knowledge of the spectral properties of natural objects is increasingly valuable for different research fields. Measured datasets of natural objects’ reflectance can offer insights into fundamental and applied research questions, contributing to investigations from coloration and color vision to color analysis and representation. Thus, datasets of natural objects’ reflectance across different locations are crucial to assessing the universality and variability of physical visual inputs in diverse environments. However, the Southern Hemisphere is notably underrepresented in publicly available datasets of natural objects. To address this gap, we present a spectral dataset of natural objects’ reflectance from the Southern cone of South America, specifically Northwestern Argentina. Our dataset encompasses 532 samples representing diverse natural objects such as barks, flowers, fruits, leaves, plant fruits, stones, and animal specimens, including birds, beetles, and butterflies. By openly sharing this dataset, as a publicly available online resource, we aim to facilitate research across various disciplines, from evolutionary biology to industrial applications.

## Background & Summary

The inquiry into the significance of coloration and color vision in animal communication and plant-animal interactions has stood as a central inquiry within the field of evolutionary biology for numerous decades^1–9^.

The spectral characteristics of light reaching photoreceptors in animals are predominantly influenced by both the spectral composition of the light itself and the surface properties of natural objects that reflect and transmit this light inhomogeneously across the optical radiant spectrum. Given substantial variations among animals in their visual systems, most notably spectral sensitivities of photoreceptors, the spectral measurement serves as a key step towards achieving a ‘receiver-independent’ assessment of an organism’s or object’s color^8,10,11^. In fact, in this frame, it is crucial to underscore that terms such as ‘coloration’ and ‘color’ denote sensory experiences rather than objective quantities.

As an exemplary case, the array of colors exhibited by flowers is extensive and has evolved under selective pressures that come from their pollinators^5,12–14^. However, it is inadequate to categorize flowers solely based on their appearance to human observers, as pollinators possess fundamentally distinct visual systems, including sensitivities to different ranges of wavelengths^15,16^. For instance, many insects have four or more spectral receptor types, with sensitivities often extending across both long and very short wavelengths^17–19^. This means that colors that appear the same to humans may appear distinct to these insects^20,21^. Indeed, visual models that estimate how animals perceive and distinguish these colors have enabled the analysis of reflectance data from their perspectives^22–25^. Therefore, databases containing natural objects’ statistics are increasingly valuable. Measurement datasets can offer insights into particular evolutionary and/or ecological questions, addressing fundamental inquiries in these (and others) fields.

In parallel, within the domain of color science, the development and assessment of novel algorithms and methodologies necessitate the validation based on high-quality spectral data^26^. These requirements are pertinent to both fundamental color research and the practical applications of color science^26^. Indeed, as highlighted by Koenderink and colleagues^27^, the effectiveness of various methodologies hinges crucially on the availability of pertinent databases. For instance, in the development of multispectral color imaging systems, insights into the spectral reflectance characteristics are highly beneficial to place a limited number of color sensors around optimal wavelength bands^28^. In this regard, it is important to tailor the appropriate dataset for a target application. Novel reflectance datasets with diverse statistical properties may improve existing applications and open avenues for novel applications.

Within this context, the measurement of spectral properties of natural objects has played a pivotal role in color and vision research. Indeed, spectral reflectance functions have been measured and analyzed for a diverse array of natural entities, including bark, flowers, fruits, grass, human skin and hair, leaves, lichen, pelage, plants, rocks, snow, soil, tree logs, and vegetation^15,21,29–40^. Furthermore, the increasing prevalence of portable spectrometers over the past two decades has facilitated the objective quantification of the spectral properties of both animal and plant structures^11,41,42^. Numerous researchers can leverage these datasets as invaluable resources to address their research inquiries. For instance, the statistical regularities uncovered by these datasets make it feasible to formulate new and plausible hypotheses regarding perceptual mechanisms and to design experimental paradigms that more accurately capture the essential characteristics of natural stimuli^43,44^. In recent years, this information has been increasingly used by machine learning algorithms in computer vision, for example, to predict material properties from spectral data^45,46^. It has been recently shown that three-dimensional perception can be improved by the use of color^47^, and color cues are important in human development stages^48^. Therefore, datasets from different regions can help to better understand different aspects of universal visual processing.

Nevertheless, it is important to acknowledge that acquiring spectral data remains a formidable task, partly because it requires specialized instrumentation for accurate measurements^26^. Consequently, publicly available datasets of natural objects are predominantly limited to specific regions worldwide, with a notable underrepresentation of the Southwestern hemisphere. The absence of datasets in a specific region hampers comprehensive characterization of the universality and variability of physical visual inputs in diverse environments. As Morimoto and colleagues^40^ pointed out, concerning visual functions at the individual level, increasing the availability of datasets across wide demographics is essential for characterizing the statistical properties of local environments to which individuals in various regions of the world are exposed.

To address the geographical bias in previous datasets and promote diversity and inclusion practices within color and vision research, the present study aimed to expand and enrich the available data and knowledge concerning the spectral properties of natural objects by constructing a spectral dataset in an underexplored geographical hemisphere. Our objective was to spectrally characterize the reflectance of a diverse array of natural objects in Argentina and to assess the distribution of chromaticities of these objects within a color space. To achieve this goal, from June 2023 to October 2024 we collected 532 samples representing 242 plant species, 63 animal taxa (comprising 68 animal specimens), and 60 rocky natural objects in Northwestern Argentina. Reflectance measurements were obtained from samples of bark (B = 20), flowers (Fl = 113), fruits (F = 31), leaves (L = 114), plant fruits (Fr = 15), vegetables (V = 9), beetles (Be = 9), birds (B = 105), butterflies (Bu = 53), and stones (S = 63).

All the measured data from our study are openly accessible in an external database. We hope that this dataset will prove to be pertinent and valuable for various research domains concerned with the spectral characteristics of natural objects, as well as for industrial applications (for instance, display technologies, as mentioned by Morimoto and colleagues^40^).

## Methods

### Collection of natural objects

To attain a comprehensive array of natural objects, we curated a diverse selection encompassing plant specimens (barks, flowers, fruits, leaves, plant fruits, and vegetables), particularly colorful animal specimens (birds, beetles, and butterflies), and stones.

The natural objects comprising our dataset were acquired through various means. The majority of items categorized as barks, flowers, leaves, and plant fruits, along with some classified as stones, were gathered within the premises of the “Centro Universitario Ing. Roberto Herrera” campus (https://maps.app.goo.gl/MHVHqmvUdPgfAjUUA), of the National University of Tucumán, and its vicinity. Fruits and vegetables, on the other hand, were procured from local fruit and vegetable markets. All animal specimens were loaned by the curators and technicians from two collections housed at the Fundación Miguel Lillo (Tucumán, Argentina; https://www.lillo.org.ar/inicio): the Ornithology Collection (Curator: Dr. Sara Bertelli; Technician: Sebastián Aveldaño) and the Entomology Collection (Curator: Mg. Emilia Pérez; Technicians: Francisco Sánchez and Walter Lemo). As for the rocky specimens, they were partly gathered from the aforementioned university campus and partly obtained from private mineral collections.

In all instances, meticulous efforts were made towards the precise identification of the natural objects. To this end, a variety of resources were consulted, contingent upon the manner of acquisition for each object. For plant specimens, scientific nomenclature was recorded using the mobile application PictureThis - Plant Identifier, developed by Glority Global Group Ltd (as Morimoto *et al*.^40^). In the case of animals, we relied upon the expertise of the personnel overseeing each collection and utilized the identifications they provided to streamline the global identification process for all natural objects. Dr. Bertelli and S. Aveldaño provided the taxonomic identification for birds, Mg. Pérez and F. Sánchez that for beetles, and Mg. Pérez and W. Lemo that for butterflies. Regarding rocky specimens, consultations were held with specialists: Dr. Sebastián Moyano, from the Facultad de Ciencias Naturales and Instituto Miguel Lillo, Universidad Nacional de Tucumán, assisted us with rocks, and Geologist Alberto Gutiérrez, from the Mineralogy Collection of the Fundación Miguel Lillo, assisted us with minerals.

### Measurement of spectral reflectance

For the measurement of spectral reflectance of natural objects, we utilized a diffuse illumination cabin designed and manufactured at the Instituto de Investigación en Luz, Ambiente y Visión (Institute of Research in Light, Environment, and Vision, ILAV), Universidad Nacional de Tucumán – Consejo Nacional de Investigaciones Científicas y Técnicas, Tucumán, Argentina. This cabin exhibits an 80% uniformity. It has dimensions of 100 cm in length, 70 cm in width, and 70 cm in height, an interior coating of matte white paint, and four incandescent lamps (type A illuminant) of 40 W/12 V, strategically positioned to minimize shadows, and powered by a controlled direct current power source.

For objects whose transparency or translucency may influence the results—specifically, certain flowers and butterflies, we introduced a black backing beneath the specimens during data collection.

The spectral reflectance measurements included in our dataset were conducted using a SpectraScan PR-715 spectroradiometer, capable of measuring the proportion of light reflected by a sample across wavelengths ranging from 380 to 1068 nm in 4 nm increments, with a 1° field of view.

Reflectance is obtained by dividing the spectrum of reflected light from the sample by the spectrum of the illuminant itself, measured using a standard sample as a reference. We used a polytetrafluoroethylene (PTFE) standard reflectance sample as the reference white for matte objects. For glossy rocky objects, we employed a glaze white ceramic tile 102 mm square with a glossy surface, specifically the CERAM Research, as the reference standard. All measurements were performed in a dark room at the Colorimetry laboratory at ILAV. Due to imprecisions in the instrument in the range of 380 nm - 396 nm and 1000 nm - 1068 nm (see Technical Validation section), we decided to only include the values from 400 nm to 1000 nm here.

**Fig. 1:**
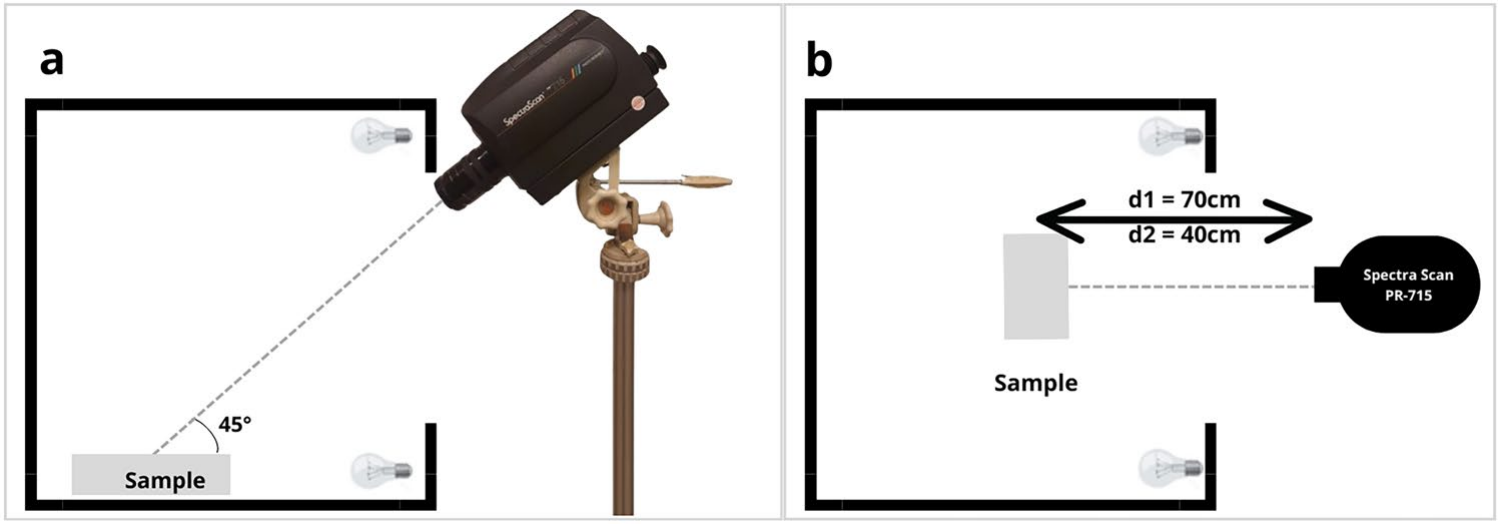
Experimental setup for the acquisition of reflectance data from natural objects. (**a**) Lateral view of the illumination cabin and the spectroradiometer, (**b**) Top view, depicting the variable distance (with two possibilities, d1 = 70 cm and d2 = 40 cm) between the spectroradiometer and the sample.

To capture the reflected light from the samples, the spectroradiometer was mounted on a tripod at an elevation angle of 45° and a variable distance (depending on the size of the sample) of either 70 or 40 cm (Fig. 1).

**Fig. 2:**
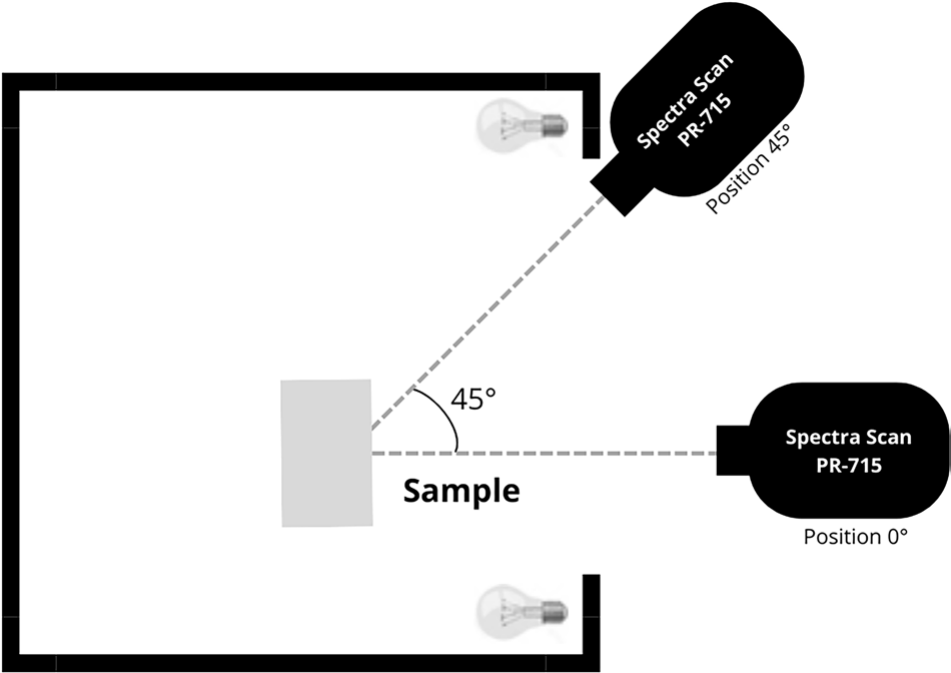
Experimental setup top view for birds and butterflies exhibiting iridescent effects on part or all of their body surface. Due to the sample size, in these cases, the distance between the spectroradiometer and the sample was fixed at 70 cm.

A special situation arises in the case of certain bird and butterfly specimens, where some colors exhibited an iridescent effect, meaning that surfaces displayed perceived colors that varied when the observer’s position changed. To take this into account in data collection, an additional variable was introduced to the experimental setup in the cases of iridescent objects, involving measurements taken with the spectroradiometer positioned at two different lateral angular orientations (at 45 and 0 degrees) relative to the sample (Fig. 2) at a unique distance of 70 cm (given the size of these samples).

**Fig. 3:**
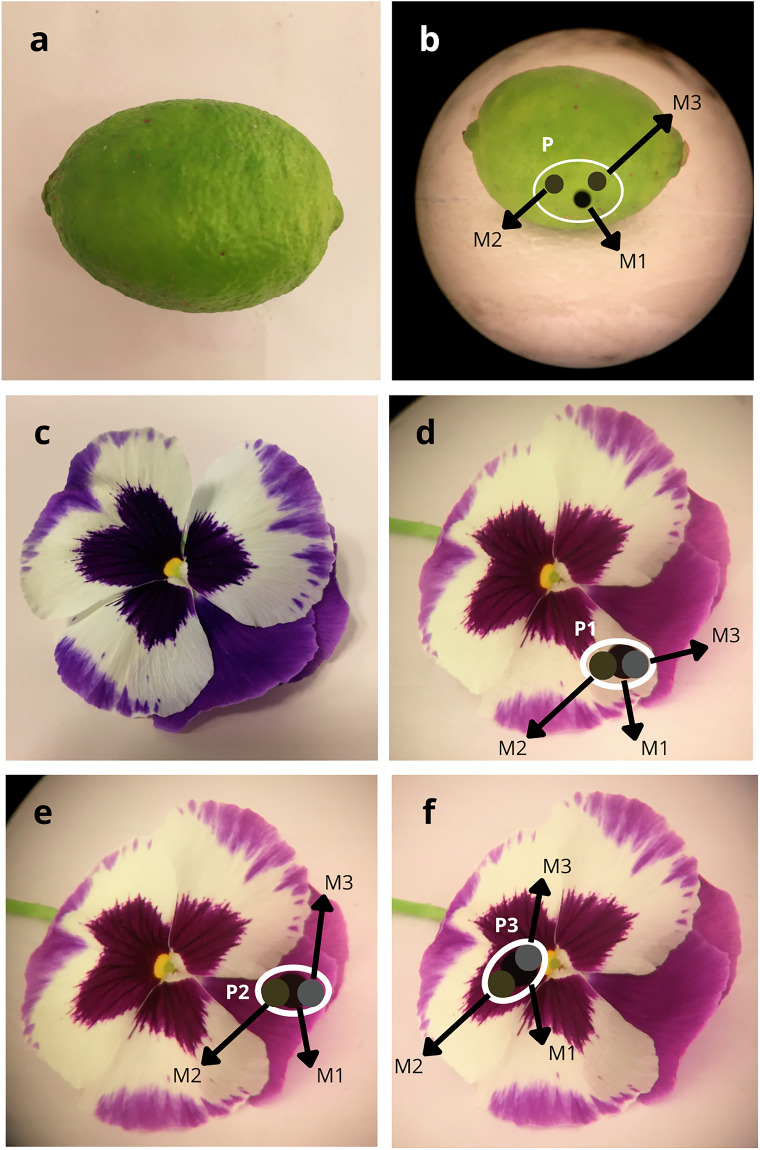
Examples of natural objects and their corresponding spectral reflectance data collection. (**a**) Specimen of *Citrus aurantifolia*, an object with a relatively simple coloration pattern. (**b**) *Citrus aurantifolia* viewed through the SpectraScan PR-715 viewer. The white outline delineates the patch to be measured, and the black and gray dots (M1, M2, and M3) represent different measurements at the same patch. (**c**) Specimen of *Viola wittrockiana*, an object with a relatively complex coloration pattern. (**d**–**f**) *Viola wittrockiana* viewed through the SpectraScan PR-715 viewer. The ellipses (P1, P2, and P3) delineate the patches (white, light purple, and dark purple, respectively) to be measured, and the black and gray dots (M1, M2, and M3) represent different measurements at each patch.

Given the wide diversity observed in the perceived colors, textures, sizes, and morphologies of the natural objects measured, we decided to measure the reflectance of the predominant observed color. In some instances, measurements were taken from multiple patches of the specimen. Reflectance measurements were repeated three times for each color patch (Fig. 3).

**Fig. 4:**
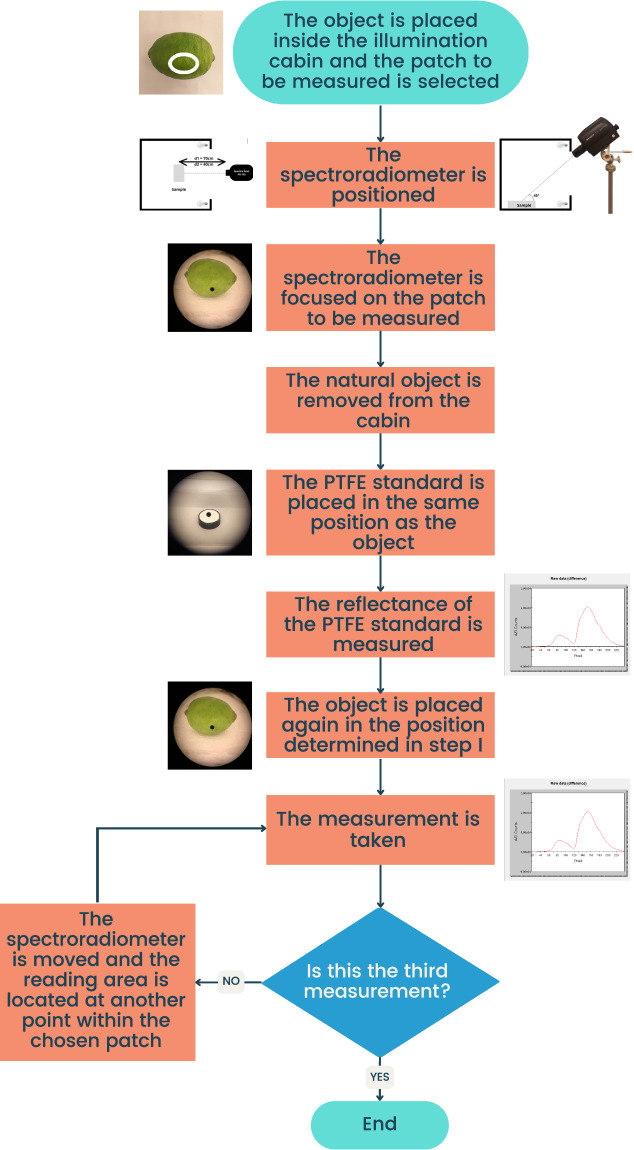
Workflow sequence for spectral reflectance data collection of natural objects.

In Fig. 4 we present a schematic summary of the workflow sequence for data collection.

### Data treatment

We classified our objects into three main domains, Plants, Animals, and Stones, and each of these was subdivided into secondary domains, namely: Barks (Ba), Flowers (Fl), Fruits (F), Leaves (L), Plant fruits (Fr), and Vegetables (V) for the “Plants” main domain; Beetles (Be), Birds (B), and Butterflies (Bu), for the “Animals” main domain; and Stones (S) for the “Stones” main domain.

**Fig. 5:**
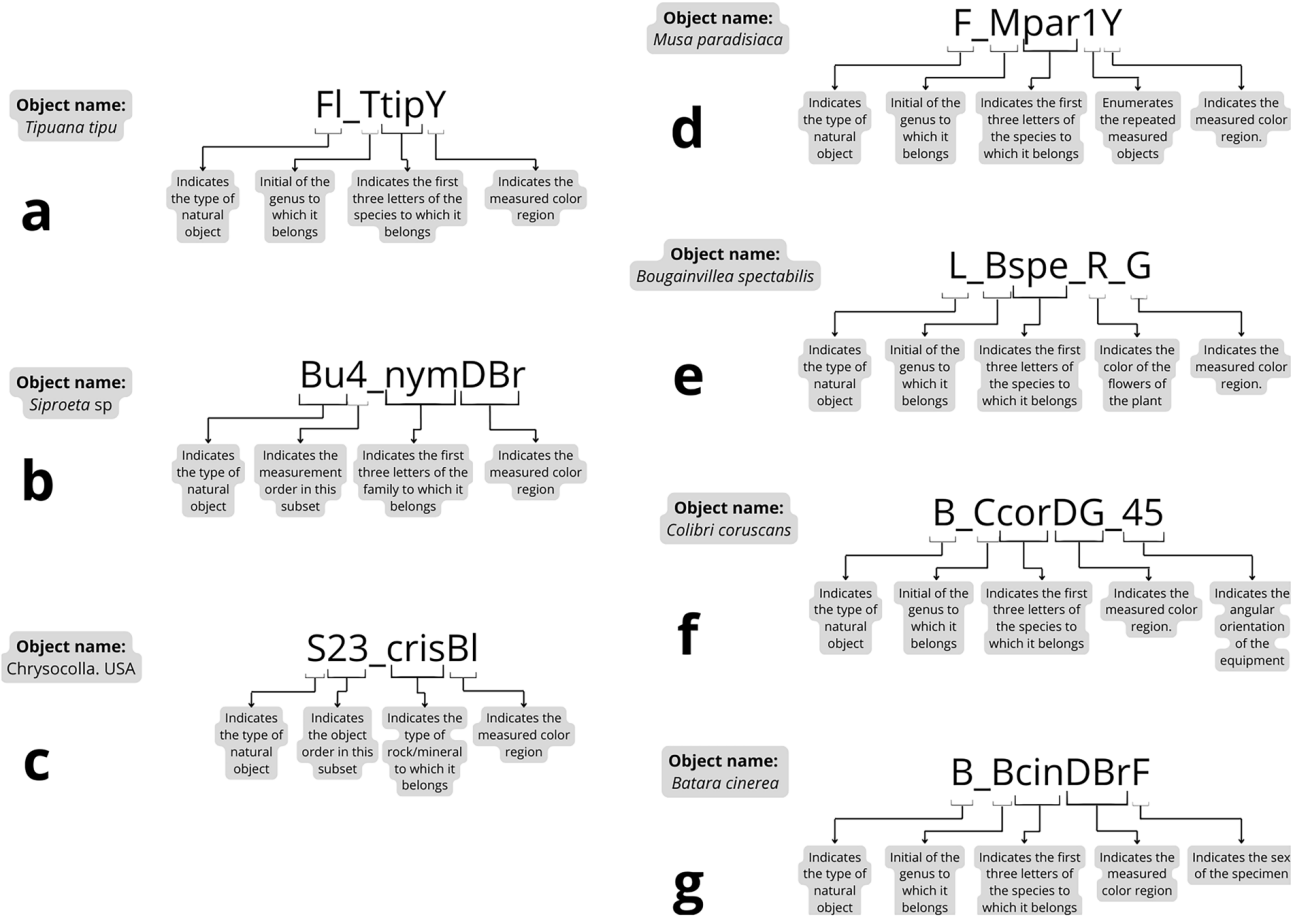
Examples of three general cases of file name structure (when different identity information was available, (**a**–**c**) and of the four exceptions to those general rules (**d**–**g**). (**a**) The case of plants and birds, for which we had a taxonomic identification at the species level. (**b**) The case of butterflies and beetles, for which we had a taxonomic identification at the family level. (**c**) The case of stones, using a general identification of the rock or the mineral. (**d**) File name structure for objects that were collected and measured more than one time (i.e., repeated objects). (**d**) File name structure for leaves that corresponded to the same plant species but with flowers of different colors. (**f**) File name structure for butterflies and birds that had iridescent colors. (**g**) File name structure for bird species that were represented for both male and female specimens.

Outputs are standardized so all our records are in the same format. To facilitate the storage, identification, and organization of resulting data files, we devised a protocol for naming each file in a manner that incorporates key information about each sample, enabling unequivocal sample retrieval. To this end, we determined that each file name should consist of the following basic components (Fig. 5a–c):

1. One or two letters indicating the secondary domain of the specimen to which the sample belongs (i.e., “Ba”, “Fl”, “F”, “L”, “Fr”, “V”, “Be”, “B”, “Bu”, and “S”); in particular cases of “Be”, “Bu”, and “S” objects, an Arabic number follows the secondary domain code because at the time of data collection, we did not have insect, rock, and mineral identities and we identified each one with those numbers;

3. An underscore;

4. Three or four letters indicating the identity of the specimen to which the sample belongs. These letters may correspond to (a) the first three letters of the family (in lowercase) when genus or species information was unavailable at the time of data collection; this applies to specimens such as beetles and butterflies; (b) the first letter of the genus (in uppercase) followed by the first three letters of the species (in lowercase); this applies to birds and all plant specimens (except for strawberries, *Fragaria* sp., which could only be identified at the genus level; hence this part of the corresponding file is named with the first letter of the genus “F” followed by “spp”); (c) the first three letters of the Spanish generic identification for rocks and minerals;

5. One or two letters indicating the perceived color of the measured patch, as follows: B (black), Bl (blue), Br (Brown), Cy (cyan), DBl (dark blue), DBr (dark brown), DG (dark green), DO (dark orange), DR (dark red), DW (dark white), DY (dark yellow), G (green), Gr (gray), LBl (light blue), LBr (light brown), LG (light green), LO (light orange), LR (light red), LY (light yellow), O (orange), P (pink), Pu (purple), R (red), W (white), Y (yellow).

There are some special cases with a few modifications for this general rule:

- Name structure for repeated objects: The file name structure for objects that were collected and measured more than one time (i.e., repeated objects), includes a number after the taxonomic information and before the color code, which enumerates the repeated measured objects (Fig. 5d).
- Name structure for leaves from plants with flowers of different colors: The file name structure for leaves that correspond to the same plant species but with flowers of different colors, includes another color code between underscores, after the taxonomic information and before the perceived color of the measured patch code, which indicates the color of the flowers of the plant to which the leaf belongs (Fig. 5e).
- Name structure for butterflies and birds with iridescent colors: The file name structure for butterflies and birds that have iridescent colors includes a number (45 or 0) after a second underscore, at the end of the name, which indicates that this patch was measured with the spectroradiometer positioned at two different lateral angular orientations relative to the sample (Fig. 5f).
- Name structure for birds with data for males and females: The file name structure for bird species that are represented for both male and female specimens includes a capital letter (F or M), at the end of the name, which indicates the sex of the specimen to which this file belongs. All files of bird specimens that have no indication of sex correspond to data from male specimens (Fig. 5g).

We present some examples of file names in Fig. 5.

## Data Records

The output dataset deposited in Figshare^49^ currently contains 532 natural objects’ reflectance spectra, each spanning the wavelength range of 400 to 1000 nm in 4 nm increments. These records correspond to plants (302 samples), animals (167 samples), and stones (63 samples; Table 1). We plan to add more records to expand the dataset shortly - particularly from plants and animals native to the study region.

**Table 1:** Total number of samples, specimens, and species (when appropriate) for each main and each secondary domain and total quantities.

Natural object category	PLANTS		
	Number of samples	Number of specimens	Number of species
Barks (Ba)	20	20	18
Flowers (Fl)	113	113	77
Fruits (F)	31	31	22
Leaves (L)	114	114	104
Plant fruits (Fr)	15	15	14
Vegetables (V)	9	9	7
**Total plants**	**302**	**302**	**242**
	**ANIMALS**		
Beetles (Be)	9	8	8
Birds (B)	105	39	36
Butterflies (Bu)	53	21	19
**Total animals**	**167**	**68**	**63**
	**STONES**		
**Natural object category**	**Number of samples**	**Number of specimens**	
Stones (S)	63	60	
**Total stones**	**63**	**60**	
**TOTAL**	**532**	**430**	

In addition to the reflectance spectral data (provided per 4 nanometer interval, no unit) from each of the three repetitions, each sample of our output dataset^49^ is associated with the following information:

- File name: File names were constructed as detailed in the Methods section. Briefly, they include the code for the corresponding secondary domain, followed by an underscore and three or four letters indicating the identity of the object, and finally, one to three letters indicating the human perceived color of the measured patch (There are some exceptions to this protocol, see the Methods section for details.).
- Measurement date: We included the date of data collection in a day/month/year format.
- Measurement angle: For iridescent colors, there were two measurement conditions according to the measurement angle, 0 or 45 degrees (see Methods for details).
- Main domain: As mentioned, we classified our natural objects into three main domains, Plants, Animals, and Stones.
- Secondary domain: In each of the main domains, we have categorized our objects into some secondary domains. For “Plants”, we included six secondary domains: Barks (Ba), Flowers (Fl), Fruits (F), Leaves (L), Plant fruits (Fr), and Vegetables (V). We grouped the “Animals” into three secondary domains, Beetles (Be), Birds (B), and Butterflies (Bu). Finally, for the “Stones”, we have only one secondary domain (Stones, S) (Table 1).
- Identity information:
  - Object name: each object has a name assigned. For the “Plants” and “Animals” main domains, it refers to the taxonomic identity of the specimen (the less inclusive taxonomic identification available). For the “Stones” domain, the object name refers to the identification of the object as a particular kind of rock or mineral.
  - Family, genus, and species (only for plants and animals): for the samples in the “Plants” and “Animals” main domains, the available taxonomic information is provided. Cells for which we do not have information (some of the taxonomic categories could not be determined for some taxa) are indicated as “N/D” (not determined). This information is not given for the “stones” main domain objects and we included the acronym “N/A” (not applicable).
- Perceived patch color: Since many natural objects have more than one color discernible to humans, we indicated as a reference the color, as perceived by researchers, of the patch from which the data was taken (see Methods for details).
- Distributional status information (for plants and animals): This information is not given for “Stones” and we included the acronym “N/A” (not applicable). For natural species of “Plants” and “Animals”, we conducted a thorough search on the internet to determine their natural distribution areas, predating any anthropogenic modifications that may have affected them. Based on this, we present the following information:
  - Distributional status: We assigned one of four categories to our biological samples: Native (when they correspond to taxa that naturally occur in environments within Argentina), Other South American (when they correspond to taxa that are naturally distributed in South American environments but their known distribution area does not include Argentina), and Exotic (when they correspond to taxa that are naturally distributed in other continents than South America). We stated a fourth category, Hybrid species, for hybrid plant species that escape the above categorization; in such cases, the term “hybrid species” does not specifically refer to their distributional status but rather to their origin from a manipulated crossbreeding of two natural species to obtain a variety with specific characteristics.
  - Source [global]: we detailed the main online freely available source considered for determining the species’ natural global distribution area.
  - Source [Argentina]: for Argentine species, we detailed the main online freely available source considered for confirming the species’ natural distribution area in Argentina.
- Other information (for plants and animals): This information is not given for “Stones” and we included the acronyms “N/A” (not applicable) for museum ID and sex, and “N/D” (not determined) for collection date and locality. For species of “Plants” and “Animals”, we provided museum ID (i.e., the number that identifies the specimen in a museum collection), the sex of the specimen, and the date and locality of the collection, when available. Cells for which we do not have information for museum ID and date and locality of the collection, are indicated as “N/A” (not available). Cells for which we do not have information for the sex of the specimen are indicated with “N/D” (not determined).

### Dataset format and characteristics

The presented dataset^49^ comprises three files corresponding to the three major groups of natural objects within our sample (plants, animals, and stones), in CSV format. This format facilitates easy access to the files while keeping their size minimal. Therefore, spectral data with metadata can be found in the CSV files “Database_Plants”, “Database_Animals”, “Database_Stones”. Explanation of how file names were generated is in the file “File name structure.txt”. The folder “Technical validation.zip” contains the data and code for validation procedures. In this folder, the data includes instrument error, PTFE reflectance, and color matching functions to obtain chromatic values and compare with existing datasets; the codes consist of Matlab codes to generate figures of measurement consistency and chromatic verification. Photographs for reference of all the samples in this dataset can be found in the file “Samples_Photographs.rar”, aiding in the visual identification of each measured object.

## Technical Validation

**Fig. 6:**
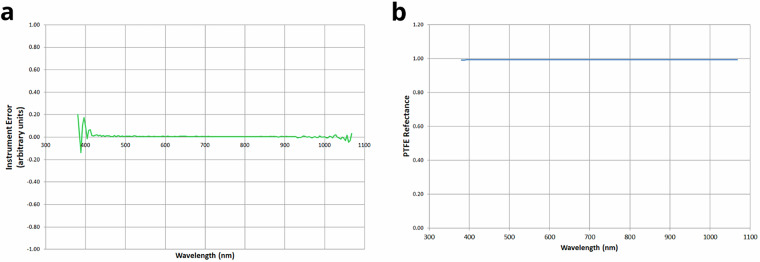
(**a**) Instrument error in arbitrary units for the SpectraScan PR-715 spectroradiometer. (**b**) PTFE reflectance as given by the manufacturer (OPTRONIC® Laboratories).

To generate our dataset^49^, we have performed the following control and calibration steps of our measurement setup:

- To ensure 80% uniformity of the diffuse lighting cabin used, 40 W/12 V type A (incandescent) lamps were connected to a current stabilizing source, maintaining the current at 3.6 ± 0.2 A.
- Instrument imprecision across wavelengths (Fig. 6a) was characterized by dividing two consecutive radiance measurements of the polytetrafluoroethylene (PTFE) standard white in the diffuse illumination cabin. Reflectance data of the PTFE standard white is shown in Fig. 6b (data provided by the manufacturer OPTRONIC® Laboratories).
- Measurements were conducted strictly following the sequence outlined in Fig. 4. In this experimental protocol, recurring measurement of the reflectance standard (either matte or glossy, as appropriate) at the same height and position of the sample being measured is of paramount importance. In all cases, the measuring instrument was aligned to look directly at the measuring plane. The optics of the measuring instruments were aligned and focused on the reflectance standard or sample, conforming to the 0°/45° geometry.
- The same equipment (specifically the SpectraScan PR-715 spectroradiometer) was used for all measurements.

### Measurement consistency

**Fig. 7:**
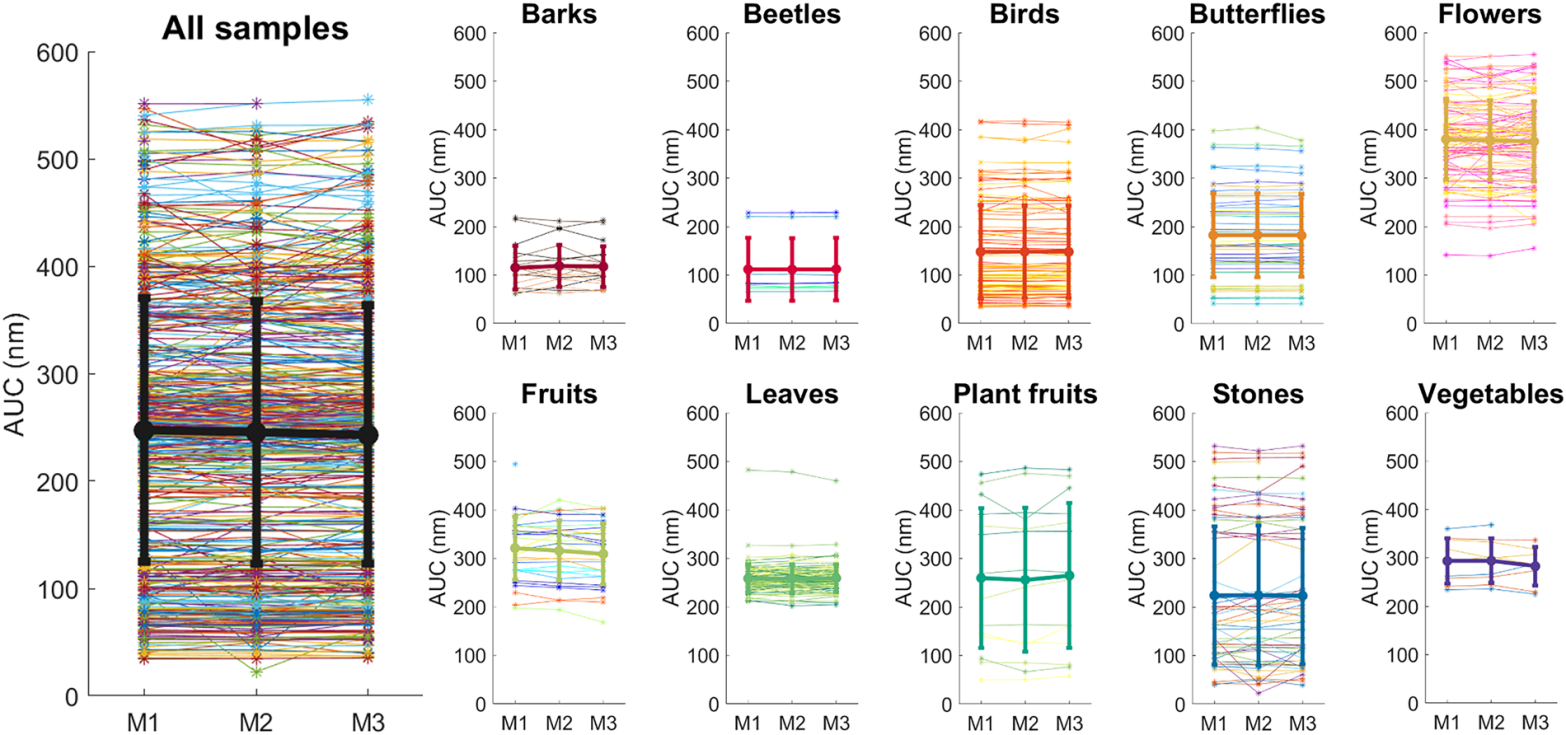
The area under the curve (AUC) values of samples’ spectra organized by category. Each panel contains the values for each sequential measurement. We found no significant differences among the three measurements [All (mixed-effects): F(1.61, 839) = 1.32, p = 0.27; Barks (rm-anova): F(1.61, 30.63) = 0.57, p = 0.54; Beetles (rm-anova): F(1.94, 15.5) = 1.17, p = 0.34; Birds (rm-anova): F(1.66, 172.3) = 0.13, p = 0.84; Butterflies (rm-anova): F(1.37, 71.47) = 1.38, p = 0.25; Flowers (mixed-effects): F(1.69, 180) = 0.93, p = 0.38; Fruits (mixed-effects): F(1.18, 33.1) = 3.89, p = 0.052; Leaves (mixed-effects): F(1.17, 131) = 0.35, p = 0.58; Plant fruits (rm-anova): F(1.69, 23.6) = 1.9, p = 0.18; Stones (rm-anova): F(1.38, 20.7) = 1.51, p = 0.24; Vegetables (mixed-effects): F(1.13, 8.49) = 0.2, p = 0.7].

Since we performed three measurements sequentially in similar regions of each object, we analyzed the agreement between these three measurements. We computed the area under the curve (AUC) for each of the three measurements for each sample (Fig. 7). Overall, the agreement is high across measurements for mean values of each category and when considering all the samples together. There were slight variations for individual samples, potentially because of variations in measured locations across the measurements. However, overall, statistical analysis verified that there is no consistent difference across measurements for mean values in each category and overall samples (detailed in the Fig. 7 caption).

### Chromatic verification

Previous datasets have identified the chromatic loci of natural objects. Thus, we further validated our dataset by comparing its chromatic gamut with that of a previous dataset collected in Japan^40^. We first averaged the reflectance across three repetitions for each sample, and then we computed the CIE1931 xy chromaticity of all 532 samples under equal energy white light.

**Fig. 8:**
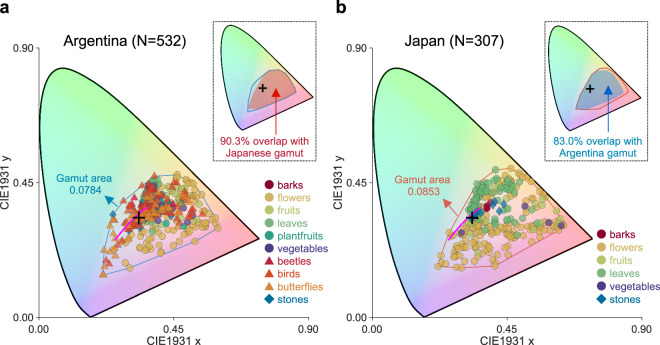
(**a**) Distribution of CIE1931 xy chromaticity of all reflectance samples collected in Argentina. Chromaticities were computed under the equal energy white light. (**b**) Chromaticity distribution of reflectance samples in Japan^40^. In both panels, the black plus shows the chromaticity of the equal energy white, and the magenta line shows the CIE daylight locus from 4,500 K to 20,000 K. Upper-right inserted figures show the chromaticity gamut and the filled region shows the overlapping region with the gamut of the other country.

Figure 8 shows the chromatic distributions of each dataset. The distributions are located around the same region in the chromaticity diagram. The gamut area for the present study (0.0784; Fig. 8a) is slightly smaller than that of the Japanese gamut (0.0853; Fig. 8b). As shown by the top-right inserted figures in each panel, both gamuts show a high percentage of overlap. Compared to the Japanese gamut, the Argentine gamut expands towards the bluish-green region, due to butterfly, bird and stone samples. In contrast, the Japanese dataset has flower samples near the spectral locus in the yellow-orange region, which are not seen in the Argentine dataset. However, the two datasets show overall similar trends despite the geographical distance between the countries, showing the high statistical regularity that might be inherent in natural objects.

## Data Availability

No custom code has been used.
